# Low Dose BMP2-Doped Calcium Phosphate Graft Promotes Bone Defect Healing in a Large Animal Model

**DOI:** 10.3389/fcell.2020.613891

**Published:** 2021-01-21

**Authors:** Tie Liu, Wen Fang, Gang Wu, Yining Li, Janak L. Pathak, Yuelian Liu

**Affiliations:** ^1^Department of Oral Implantology, The Affiliated Hospital of Stomatology, School of Stomatology, Zhejiang University School of Medicine, Hangzhou, China; ^2^Key Laboratory of Oral Biomedical Research of Zhejiang Province, Hangzhou, China; ^3^Department of Periodontology, The Affiliated Hospital of Stomatology, School of Stomatology, Zhejiang University School of Medicine, Hangzhou, China; ^4^Department of Oral Implantology and Prosthetic Dentistry, Academic of Dentistry Amsterdam (ACTA), VU Universiteit Amsterdam and University of Amsterdam, Amsterdam, Netherlands; ^5^Department of Oral Pathology, The Affiliated Hospital of Stomatology, School of Stomatology, Zhejiang University School of Medicine, Hangzhou, China; ^6^Guangzhou Key Laboratory of Basic and Applied Research of Oral Regenerative Medicine, Affiliated Stomatology Hospital of Guangzhou Medical University, Guangzhou, China

**Keywords:** bone regeneration, BioCaP graft, BMP2, osteoinduction, bone defect healing

## Abstract

**Background:** Bone grafts are in high demand due to the increase in the cases of bone defects mainly caused by trauma, old age, and disease-related bone damages. Tissue-engineered calcium phosphate (CaP) biomaterials match the major inorganic contents of bone, thereby could be the potential bone graft substitute. However, CaP-bone grafts lack the osteoinductivity that is vital for effective bone regeneration. In this study, we aimed to test the bone defect healing potential of biomimetically fabricated low dose BMP2-doped CaP (BMP2.BioCaP) grafts in a large animal model.

**Methods:** Low dose BMP2 was doped internally (BMP2-int.BioCaP) or on the surface of CaP (BMP2-sur.BioCaP) grafts during the fabrication process. Our previous study showed the robust bone regenerative potential of BMP2-int.BioCaP and BMP2-sur.BioCaP grafts in the rat ectopic model. In this study, we investigated the bone defect healing potential of BMP2.BioCaP grafts in sheep humerus/femoral defects, as well as compared with that of autologous bone graft and clinically used deproteinized bovine bone (DBB) xenograft.

**Results:** Different ways of BMP2 doping did not affect the surface morphology and degradation properties of the graft materials. Micro-CT and histology results showed robustly higher bone defect-healing potential of the BMP2.BioCaP grafts compared to clinically used DBB grafts. The bone defect healing potential of BMP2.BioCaP grafts was as effective as that of the autologous bone graft. Although, BMP2-int.BioCaP doped half the amount of BMP2 compared to BMP2-sur.BioCaP, its' bone defect healing potential was even robust. The BMP2.BioCaP grafts showed less immunogenicity compared to BioCaP or DBB grafts. The volume density of blood vessel-like and bone marrow-like structures in both BMP2.BioCaP graft groups were in a similar extent to the autologous group. Meticulous observation of higher magnification histological images showed active bone regeneration and remodeling during bone defect healing in BMP2.BioCaP graft groups.

**Conclusion:** The robust bone regenerative potential of BMP2.BioCaP grafts in the ectopic model and *in-situ* bone defects in small and large animals warrant the pre-clinical studies on large animal critical-sized segmental bone defects.

## Introduction

Large bone defects caused by trauma, fracture, tumor resection, infection, and congenital malformation substantially impact patient health and quality of life (Greenwald et al., 2001; Huang et al., 2016; Kumar et al., 2016). The global bone graft substitutes market was valued at US$ 2.9 Bn in 2019 and is anticipated to expand at a Compound Annual Growth Rate of ~3% from 2020 to 2030 (Transperancy Market Research Report, 2020). Bone defect reconstruction is a complex biological process that involves proper bone grafts, a supply of precursor cells and growth factors, vascularization, immunomodulation, and osteogenesis (Greenwald et al., 2001; Huang et al., 2016). Therefore, the reconstruction of large bone defects is still a challenge for orthopedic and craniomaxillofacial surgeons. To treat a large bone defect, a functional bone graft must be implanted. Autografts are regarded as the gold standard for repairing bone defects; (Jung et al., 2003; Ferreira et al., 2013) however, their application is limited due to low graft availability and donor site morbidity (Nkenke et al., 2004). Alternative bone grafts such as allografts and xenografts are clinically used for critical size bone defect healing. Limited source, immunotoxicity, and risk of infection are the main limitations of these alternative bone grafts (Van Der Stok et al., 2011; Sun et al., 2019). Bone tissue engineering approaches are focused on developing novel biomaterials that address the limitations of conventional autogenic, allogenic, or xenogenic bone grafts.

The tissue-engineered ideal bone graft should be biocompatible, osteoconductive, osteoinductive, angiogenic, non-immunogenic, and biodegradable (Haugen et al., 2019). Recent advances in bone tissue engineering had developed 3D-prinetd biomaterial-based patient-specific scaffolds with enhanced biological and mechanical properties (Zhang et al., 2019). Synthetic scaffolds in combination with bioactive molecules or stem cells has shown promising potential for large size bone defect healing (Ho-Shui-Ling et al., 2018). Bone ingrowth pattern in synthetic scaffold directly correlates with the pore scale morphology and physical properties of the scaffolds (Jones et al., 2009). The primary inorganic component of bone is calcium phosphate (CaP), mainly the hydroxyapatite. CaP-based biomaterials including hydroxyapatite and beta-tricalcium phosphate had shown promising potential to be used as a bone graft (Samavedi et al., 2013). So far developed CaP-based bone grafts are reported to be biocompatible and osteoconductive, but they lack osteoinductivity. Doping osteogenic and/or angiogenic growth factors such as BMP2, VEGF, or FGF in biomaterials improves the bone regenerative potential of the bone grafts (Chim et al., 2013; Chen R. et al., 2016). Emerging pieces of literature had reported a robust bone defect healing potential of BMP2-loaded biomaterials (Selvig et al., 2002; Rao et al., 2013; Oortgiesen et al., 2014; Halloran et al., 2020). Although the Food and Drug Administration (FDA) approved usage of recombinant human BMP-2 (rhBMP-2) during spinal fusion surgery, tibial shaft repair, and maxillary sinus reconstructive surgery, series of systemic adverse effects were experienced during clinical application (Zhang et al., 2015; Halloran et al., 2020). Such systemic adverse effects of BMP2 applied for bone regeneration are mainly due to the burst released high dose BMP2 (James et al., 2016). Reducing the dose of doped BMP2 in graft materials and maintaining the slow and sustained release of low dose BMP2 in the defect site could minimize the systemic adverse effects of the BMP2. Moreover, the traditional CaP bone graft materials lack biomimicry and physicochemical properties required for bone defect healing (Fernandez-Yague et al., 2015). To overcome the above-mentioned issues, we had developed the BMP2-doped biomimetic CaP (BMP2.BioCaP) granules as a bone graft substitute with a robust bone defect healing potential (Liu et al., 2017; Wang et al., 2019).

The bone defect healing potential of the majority of the artificial bone grafts is usually tested in small animal models, which cannot represent the exact bone remodeling and bone properties in the human body. Large animal models, including sheep, dogs, and pigs, provide insights on the bone defect healing potential of artificial bone grafts in tibial and femoral bone defects with relevant clinical similarities to human bone defects (Weigand et al., 2017; Mcgovern et al., 2018). However, the differences in types of bone defects, animal models, surgical methods, and fixation procedures used in literature to test bone defect healing potential of bone grafts make it difficult to draw conclusions for clinical translation (Reichert et al., 2012). Among the large animal models, sheep bone properties and bone-remodeling rate are comparatively close to the human bone (Mcgovern et al., 2018; Hettwer et al., 2019). Moreover, sheep are less aggressive and easy to handle and house (Pearce et al., 2007; Reichert et al., 2012; Ingavle et al., 2019). Our previous studies had shown the bone defect healing potential of BMP2.BioCaP grafts alone or in combination with DBB in ectopic and critical size bone defects in murine models (Liu et al., 2014; Wang et al., 2017, 2019). Interestingly, BMP2.BioCaP grafts in the combination of DBB healed sheep humeral/femoral bone defect as effectively as the autologous bone graft (Liu et al., 2017). However, the bone defect healing potential of BMP2.BioCaP bone grafts alone in a large animal model has not been investigated yet.

**Figure 1:**
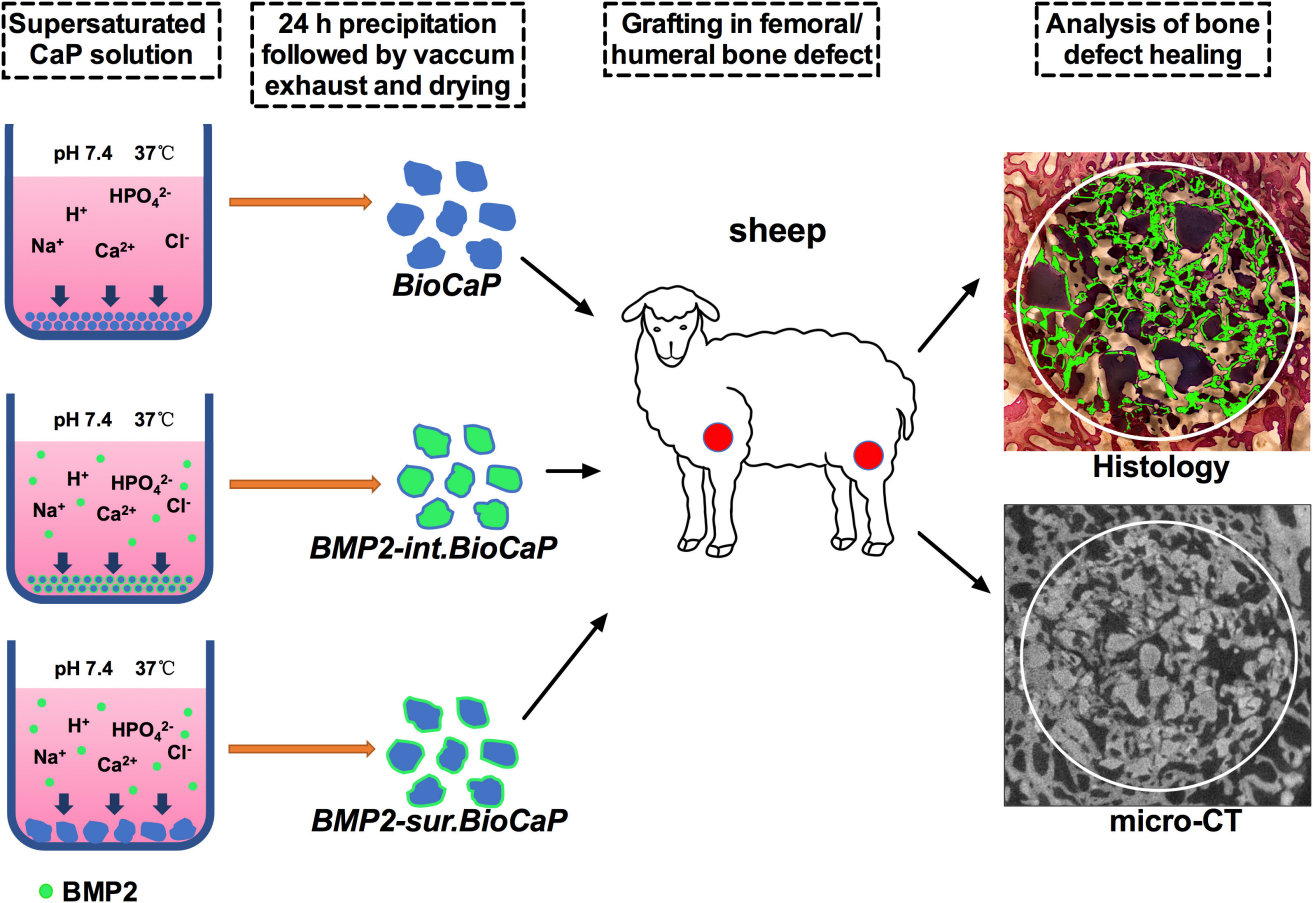
Scheme of BMP2.BioCaP preparation and sheep femoral/humeral bone defect healing potential analysis.

Different methods of BMP2-doping incorporate the distinctive concentrations of BMP2 in bone grafts and affect the bioactivity of the grafts contrarily. We had developed BioCaP with an internal or surface biomimetic coating of BMP2. The internal or surface biomimetic coating of BMP2 osteogenically functionalizes the BioCaP grafts as indicated by the robust bone regeneration in ectopic transplantation (Liu et al., 2014). Moreover, BMP2 release from the bone graft is mainly controlled by the cellular activity around the graft. In this study, we aimed to further investigate the bone defect healing potential of BMP2.BioCaP grafts in a large animal model (sheep) as indicated in Figure 1. We also compared the bone defect-repairing efficacy of BMP2.BioCaP grafts with that of autologous bone graft and clinically used deproteinized bovine bone (DBB) xenograft. BMP2.BioCaP grafts (BMP2 internally or on surface coating) promoted bone defect healing in sheep as effectively as the autologous graft.

## Materials and Methods

### Fabrication of Biomimetic Calcium Phosphate (BioCaP) Granules

Since the incubation of biomaterials in 5-fold concentrated simulated body fluid can deposit a CaP layer resembling the *in vivo* surface structure, this method is named as a biomimetic coating (Lin et al., 2020). BioCaP granule was fabricated by refining a well-established biomimetic mineralization process (Wang et al., 2019). Briefly, a CaP solution (200 mM HCl, 20 mM CaCl_2_·2H_2_O, 680 mM NaCl, and 10 mM Na_2_HPO_4_) buffered by TRIS (250 mM) to a pH of 7.4 was incubated in a shaking water bath (50 agitations/min) at 37°C for 24 h. For sterilization, the CaP solution was filtered with the vacuum filter (0.22-μm pore) before buffering. Precipitation was retrieved and gently washed by Milli-Q water, strongly filtered and compressed to a block using a vacuum exhaust filtering method with a vacuum filter (0.22-μm pore, Corning, NY, USA) and an air pump. After drying in air circulation at room temperature for 2 h, the hardened block was ground and filtered through metallic mesh filters to obtain BioCaP granules with a size of 0.25–1.0 mm. All the procedures were performed under aseptic conditions.

### Biomimetic Coating to Fabricate BMP2.BioCaP Grafts

BioCaP granules are osteoconductive but lack osteoinductive properties. A low dose of BMP2 was incorporated in BioCaP granules to obtain osteoconductive and osteoinductive BMP2.BioCaP graft materials. BMP-2 (INFUSE® Bone Graft, Medtronic, USA) was introduced into the CaP solution at a final concentration of 0.2 μg/ml before buffering and co-precipitated into the interior of BioCaP (BMP2-int.BioCaP), viz. the internally-incorporation mode. The doses of BMP2 and CaP solution were determined based on the results of our previous studies (Liu et al., 2014; Wei et al., 2019).

The superficial BMP2 coating was deposited on BioCaP granules according to the well-established biomimetic mineralization approach to obtain BMP2-sur.BioCaP (Liu et al., 2014). Briefly, 0.58 g of BioCaP granules (size: 0.25–1 mm) was incubated in the coating solution [40 mM HCl, 4 mM CaCl_2_·2H_2_O, 136 mM NaCl, 2 mM Na_2_HPO_4_, and 50 mM TRIS (pH 7.4); total volume of 150 ml] containing 1 μg/ml BMP2 in a shaking water bath (50 agitations/min) at 37°C for 24 h.

### Quantification of Incorporated BMP-2

The amount of incorporated BMP-2 was determined by a commercially available BMP2 enzyme-linked immunosorbent assay (ELISA) kit (PeproTech, London, UK). BMP2-int.BioCaP and BMP2-sur.BioCaP (0.05 g/sample) were dissolved in 1 ml 0.5 M EDTA (pH 8.0) for 10 min in a 100-rpm stirrer. The ELISA assay was performed according to the manufacturer's instructions (*n* = 6/group). The BioCaP without BMP2 incorporation was used as a control.

### Surface Characteristics and Protein Distribution in BioCaP Grafts

The morphology and surface characteristics of BMP2.BioCaP grafts were visualized in a scanning electron microscope (SEM, XL20, FEI Company, the Netherlands), under an accelerating voltage of 10 kV using sputter-coated with gold. To analyze the pattern of BMP2 protein distribution in BMP2.BioCaP, FITC-BSA was used as a mimic of BMP2 to incorporate in BioCaP and visualized under a fluorescence microscope as described previously (Liu et al., 2014).

### *In vivo* BMP2.BioCaP Grafting in Bone Defect of Large Animal Model

Twelve Australian sheep (4 years old female, 40–50 kg body weight) were purchased from the Military Veterinary Institute, Quartermaster University of PLA, Changchun, China and housed at the Animal facility of Zhejiang University, Zhejiang, China. This study adapted the 4 years age sheep and defect model based on the previous literature (Nuss et al., 2006; Kobayashi et al., 2010; Liu et al., 2017). Sheep were anesthetized by administering Sumianxin II (0.3 ml/kg,) with the addition of penicillium (5 × 10^4^ U/kg) and atropine (0.03 mg/kg) at 30 min before surgery. Local anesthesia (1% lidocaine with 1:100,000 adrenaline) and skin disinfection (0.5% iodophor solution) were applied to the proximal part of femoral diaphysis or proximal diaphysis of humerus or distal epiphysis of humerus. Six implantation sites/sheep were randomly chosen. Cylindrical defects (8 mm diameter and 13 mm deep) were created in the cancellous bone anatomical sites (proximal part of femoral diaphysis or proximal diaphysis of humerus or distal epiphysis of humerus) as described previously (Nuss et al., 2006; Liu et al., 2017). These implantation sites were assigned to the six experiment groups according to a randomization protocol (Wang et al., 2012). A total of 72 defect areas were used in this study with 36 each for 4 and 8 weeks (6 defects/group). This study was approved by the Ethical Committee of the School of Stomatology, Zhejiang University. All the animal experiments were carried out according to the ethics laws and regulations of P.R. China. Throughout the study, the sheep were treated following the ARRIVE guidelines. The following six groups were established to analyze the critical-sized bone defect healing potential of BMP2.BioCaP grafts.

(i) No graft (Negative control)
(ii) Autologous bone graft (Positive control)
(iii) DBB graft, bovine bone (Bio-Oss®) (Clinical standard control)
(iv) BioCaP graft
(v) BMP2-sur.BioCaP graft
(vi) BMP2-int.BioCaP graft

The amount of graft and incorporated BMP2 used in each group is elaborated in Table 1. In the case of autograft, the autologous bone was harvested during the creation of the defect and minced to 0.25–1 mm chips using a rongeur. After implantation of grafts, the Geistlich Bio-Gide® membrane (Geistlich Biomaterials, Wolhuser, Switzerland) was used to cover the defect. All sheep were allowed to walk in the confined plain grassland. One week after surgery all the sheep showed normal mobility. All the sheep exhibited good health and all the surgical implant sites healed well without any significant wound complication. No visual signs of inflammation or adverse host tissue reaction were observed. After 4 or 8 weeks of grafting, sheep were sacrificed by an overdose of intramuscular veterinary Sumianxin II (Jilin Huamu Animal Health Product, Jilin Province, China) injection. Bone grafts with surrounding tissues were retrieved and processed for histological and micro-CT analysis.

**Table 1:** Graft materials implanted in different experimental groups.

**Group**	**Graft material (volume/graft)**	**Loaded BMP2/graft**
(i) No graft	-	-
(ii) Autologous bone	0.65 cc	
(iii) DBB	0.65 cc (0.35 g)	-
(iv) BioCaP	0.65 cc (0.58 g)	-
(v) BMP2-sur.BioCaP	0.65 cc (0.58 g)	19.28 μg
(vi) BMP2-int.BioCaP	0.65 cc (0.58 g)	9.95 μg

### Micro Computed Tomography (Micro CT) Imaging and Quantification

Retrieved grafts with surrounding tissues were fixed in a 10% neutral buffered formalin solution and embedded into an MMA block as described previously (Liu et al., 2005; Wu et al., 2011). Three-dimensional distribution of BMP2.BioCaP graft with bone tissues in the bone defects was measured with a high-resolution micro CT system (μCT 40, Scanco Medical AG, Brüttisellen, Switzerland). Each specimen of the bone defect cylinder was positioned vertically and scanned with an isotropic spatial resolution of 18 μm and the beam energy 70 kV source voltage, 113 μA current, and an 18 μm isotropic voxel size. The specimens were mounted in cylindrical specimen holders (polyetherimide; outer diameter: 35.0 mm, wall thickness: 1.0 mm) and secured with synthetic foam to make sure the bone defect cylinder was vertical. The integration time of 750 ms was used to minimize noise. The system was equipped with an aluminum filter and a correction algorithm to minimize beam-hardening artifacts (Mulder et al., 2006).

The gray values of each specimen, which depends on the radiopacity of the scanned material were converted into the degree of mineralization with the analysis software (Scanco Medical AG). Newly formed bone was distinguished from bone graft materials using the novel “onion-peeling” algorithm (Scanco Medical AG) (Schulten et al., 2013). Briefly, a low threshold of 560 mg hydroxyapatite (HA)/cm^3^ was used to discriminate bone tissue from connective tissue and bone marrow. The gray values were scaled from 1 to 1,000 and the threshold was set at 200 to distinguish bone graft materials from newly formed bone tissue. These two thresholds were calculated by averaging the thresholds resolved in three slices of three samples by two blinded observers. Using this approach, we measured the bone volume/total volume in the bone grafts.

### Histological Procedures

Retrieved grafts with surrounding tissues were fixed and embedded into an MMA block as described previously (Liu et al., 2005; Wu et al., 2011). The tissue embedded blocks were trimmed and sawn vertically to the long axis into 10–12 slices of 600 μm thickness at an interval of 1 mm applying a systematic random-sampling strategy (Gundersen and Jensen, 1987). Slices of each sample were separately mounted on plexiglass holders and polished. The slices were surface-stained with McNeal's tetrachrome, basic fuchsine, and toluidine blue (Wu et al., 2011) and examined with a light microscope with a digital camera (Leica, Wetzlar, Germany).

### Histomorphometric Analysis

In addition to a subjective histological description, 10 slices of each graft were used for quantitative histomorphometric analysis including the volume of newly formed bone, bone marrow-like structure, remaining BioCaP, and the volume density of multinucleated giant cells (MGCs). The surface area (S) of a component per slice was obtained by two blinded observers using the point-counting technique in the whole area of 8 mm diameter of each slice (Cruz-Orive and Weibel, 1990). The interval between the two slices was 1 mm. Therefore, the volume (V) of a component is defined as V = $\overset{10}{\underset{n=1}{\sum }}\left(Sn\times 1\right)$.

The volume density of MGCs was normalized to the volume of BioCaP or DBB. The volume density of MGCs (Va) is defined as its volume (Vb) per unit volume of graft materials (Vc): Va = Vb/Vc. To evaluate the degradation of BioCaP granules, the volume of BioCaP before implantation (time 0, as control) was evaluated by the same histological method. Six chemically fixed and plastic-embedded samples (0.58 g of BioCaP granules per sample) were specifically reserved for this purpose.

### Statistical Analysis

All data are presented as mean ± standard deviation (SD). Data were compared using one way analysis of variance (ANOVA), and Bonferroni's correction was used for *post-hoc* comparison. The significance level was set at *p* < 0.05.

## Results

### *In-vitro* Characterization

**Figure 2:**
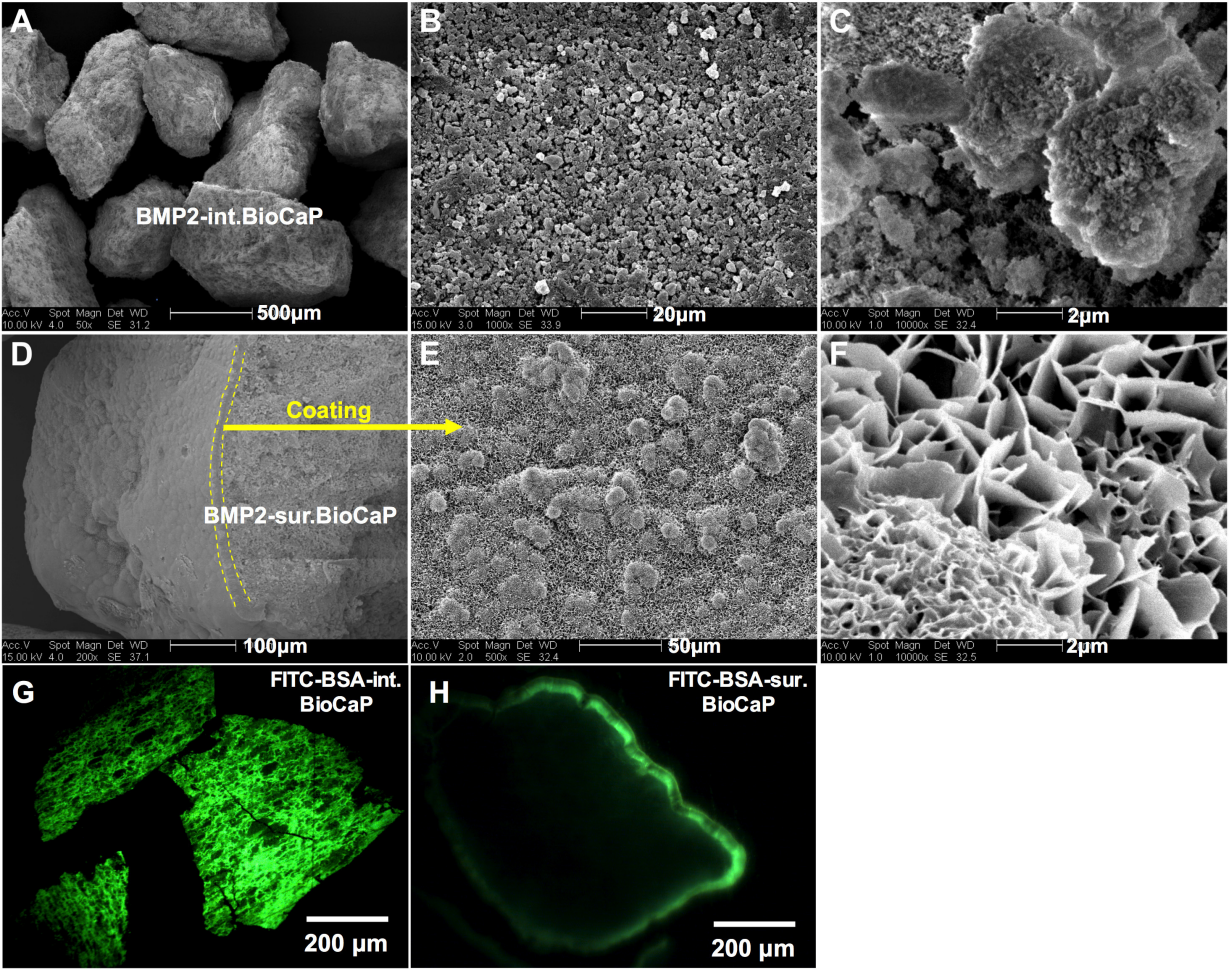
Surface characteristic and morphology of BMP2.BioCaP grafts. Representative SEM images of BMP2-int.BioCaP **(A–C)** and BMP2-sur.BioCaP **(D–F)** from lower to higher magnification. Representative fluorescence images of FITC-BSA-int.BioCaP **(G)**, and FITC-BSA-sur.BioCaP **(H)**, showing the protein loading and distribution pattern.

BMP2.BioCaP grafts with random shape and 0.25–1.00 mm size were successfully synthesized. BMP2-int.BioCaP showed a homogeneous rough surface (Figures 2A–C). BMP2-sur.BioCaP showed the crystalline surface (Figures 2D–F). SEM images indicate that the biomimetic technique of BMP2 incorporation in BioCaP to develop BMP2.BioCaP graft did not affect the surface morphology (Figures 2A,F). The internal protein incorporation technique ensured the homogenous distribution of protein (green color) in the outer and inner part of the BioCaP (Figure 2G). In surface protein incorporated BioCaP, the protein was localized only on the periphery of the graft (Figure 2H). ELISA results indicated that BMP2-sur.BioCaP loaded 2 times higher dose of BMP2 (19.28 μg vs. 9.95 μg) compared to BMP2-int.BioCaP graft (Table 2).

**Table 2:** Amount of BMP2 loaded in two type of BMP2.BioCaP grafts.

**Sample number**	**Loaded BMP2 in BMP2-sur.BioCaP/graft (μg)**	**Loaded BMP2 in BMP2-int.BioCaP/graft (μg)**
1	19.89	10.03
2	19.90	9.40
3	20.08	11.35
4	19.10	9.30
5	18.84	8.20
6	17.90	11.40
Average	**19.28**	**9.95**

### Micro-CT Analysis

**Figure 3:**
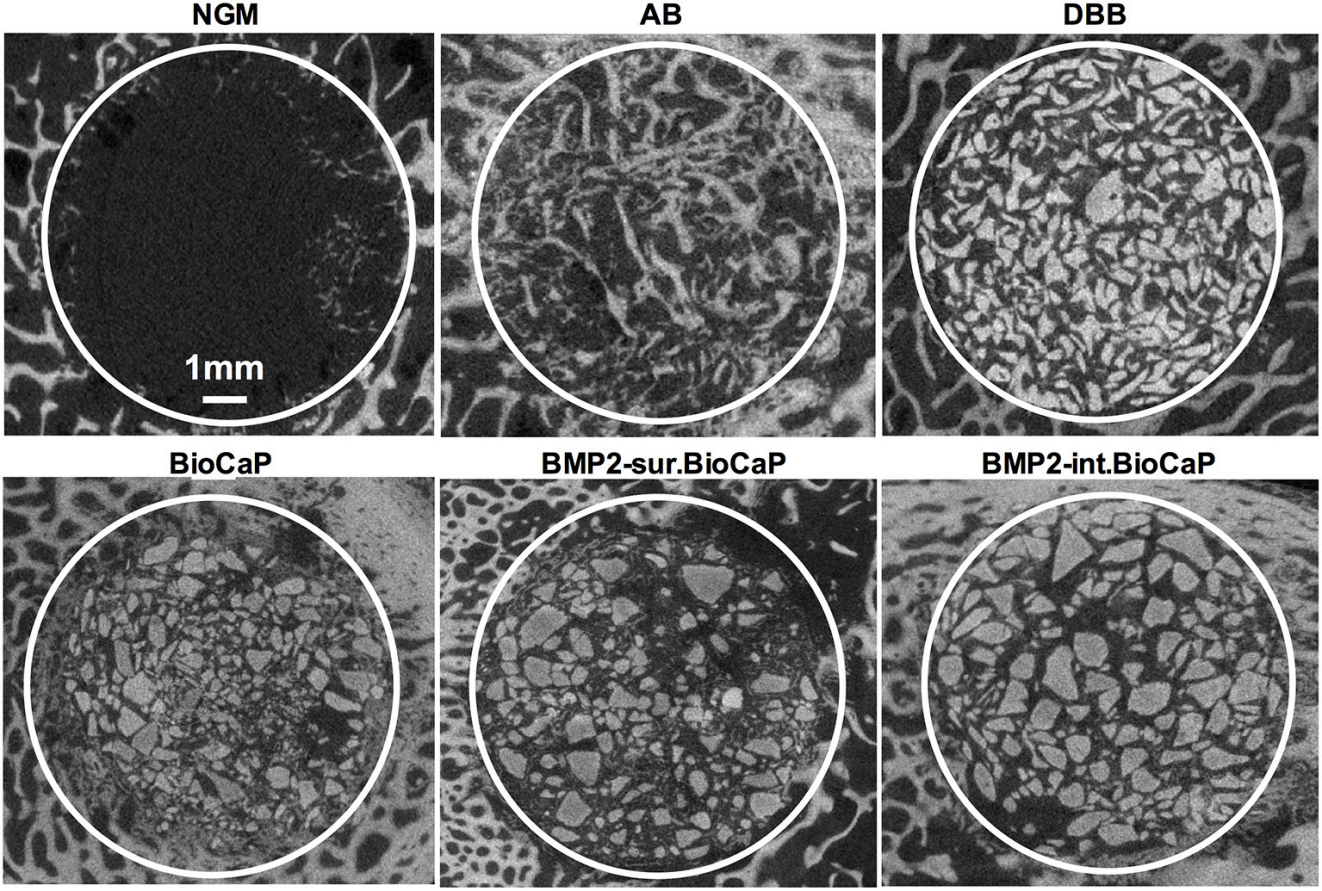
Micro-CT images showing newly formed bone and grafted biomaterials in the defect site after 4 weeks of grafting.

**Figure 4:**
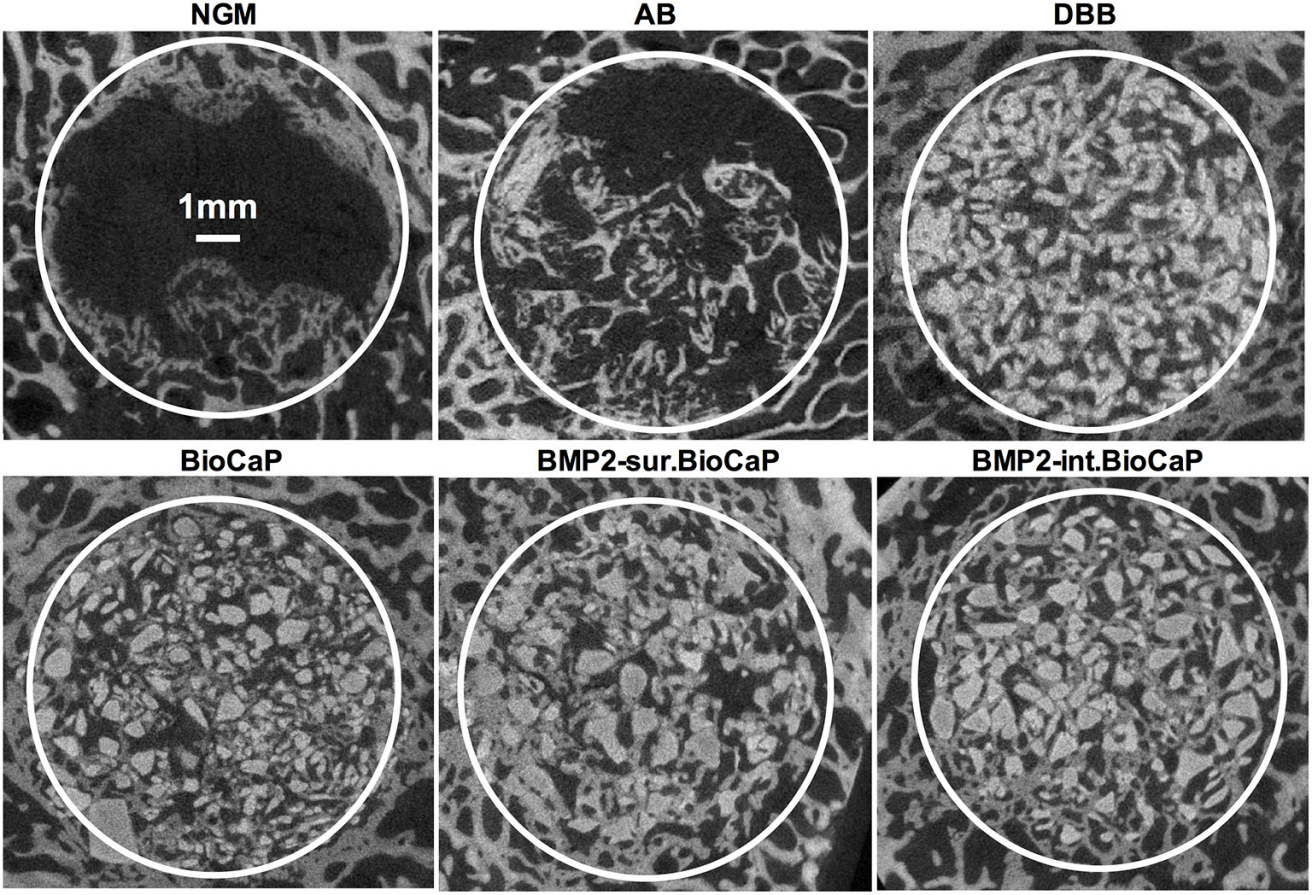
Micro-CT images showing newly formed bone and grafted biomaterials in the defect site after 8 weeks of grafting.

Figure 3 shows the micro-CT images of bone defects after 4 weeks of surgery. An almost empty defect was observed in the no graft group. The homogeneous distribution of graft materials was observed in all other groups (Figure 3). Figure 4 shows the micro-CT images of bone defects after 8 weeks of surgery. The newly formed bone was observed in the periphery of the no graft group. In the autologous bone graft group, newly formed bone was observed in the center and periphery with some empty area. This empty area might be the result of faster degradation properties of autologous bone graft. In the synthetic bone graft groups, the faded white color indicates the newly formed bone and the sharp white color indicates the remaining graft materials (Figure 4). In DBB and BioCaP group, the defect area was mainly covered by graft materials and with a meager amount of newly formed bone. BMP2-sur.BioCaP and BMP2-int.BioCaP group showed a comparatively bigger area of newly formed bone.

### Histological Analysis

**Figure 5:**
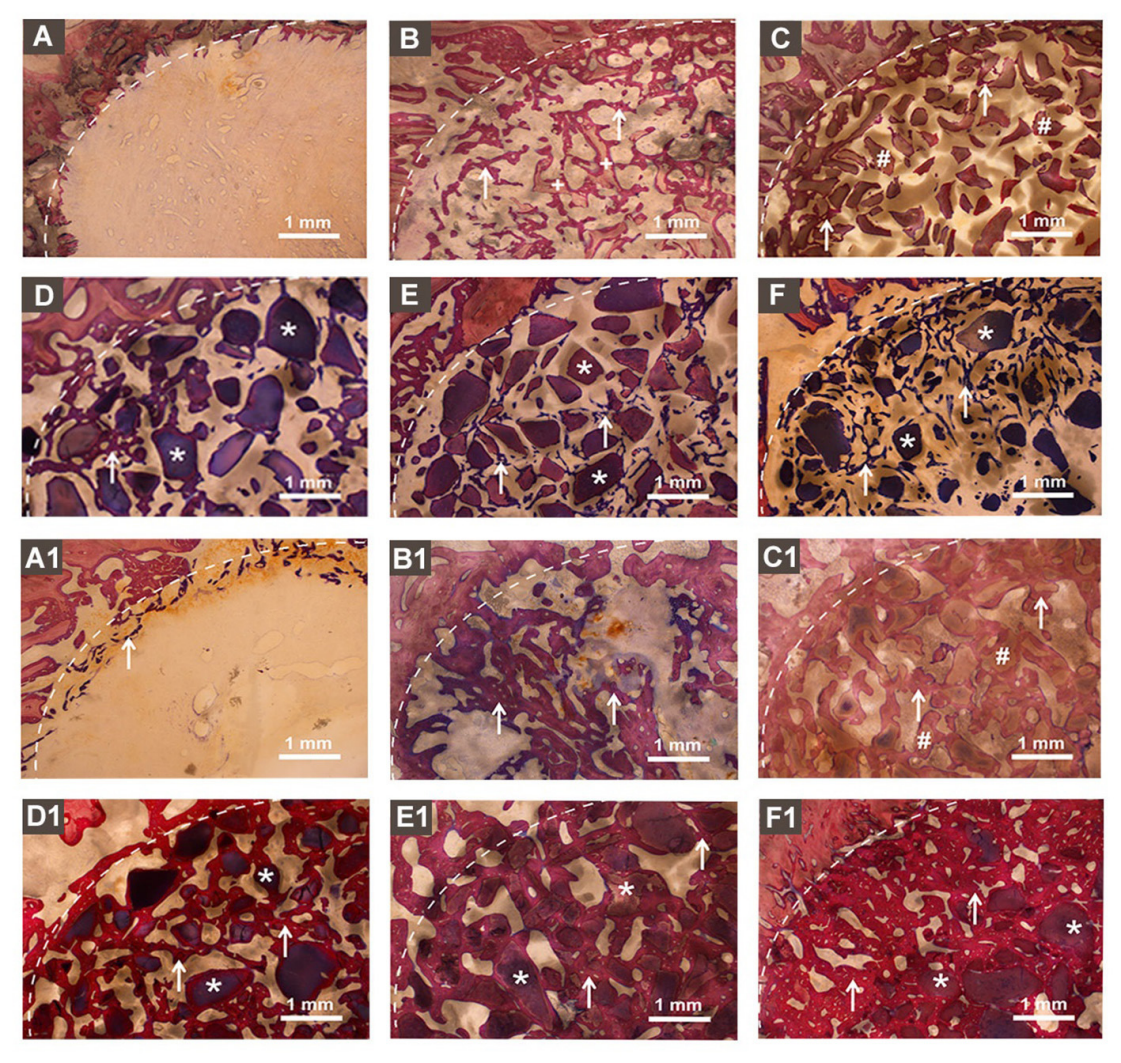
Histological images in showing newly formed bone and remaining bone grafts. **(A)** no graft, **(B)** autologous bone, **(C)** DBB, **(D)** BioCaP, **(E)** BMP2-sur.BioCaP, and **(F)** BMP2-int.BioCaP after 4 weeks of grafting. **(A1)** no graft, **(B1)** autologous bone, **(C1)** DBB, **(D1)** BioCaP, **(E1)** BMP2-sur.BioCaP-group, and **(F1)** BMP2-int.BioCaP after 8 weeks of grafting. White dot line: demarcation between native bone and newly formed bone; white stars: remaining graft materials; +: remaining autologous bone; #: remaining DBB.

Figures 5A–F represents the McNeal's tetrachrome, basic fuchsine, and toluidine blue stained histological images of tissue sections from bone defects at week 4. Histological images showed an empty defect in no graft group (Figure 5A). The newly formed bone surrounding the autologous graft was observed in the autologous bone graft group (Figure 5B). The newly formed bone was hardly observed in the DBB and BioCaP group (Figures 5C,D). Homogeneous distribution of newly formed bone surrounding the graft and in space between graft materials was observed in BMP2-sur.BioCaP and BMP2-int.BioCaP group. Figures 5A1–F1 represents the histological images of tissue sections from bone defects at week 8. Histological images from 8 weeks showed a higher amount of newly formed bone in all the groups compared to images from 4 weeks of the respective group (Figures 5A1–F1). This result indicates the active bone regeneration process during bone defect healing. Newly formed bone was spotted only in the periphery of the defect in no graft group (Figure 5A1). The autologous bone graft group mainly showed newly formed bone with almost no remaining graft materials (Figure 5B1). Big areas of empty spaces were noticed in the autologous bone graft group, possibly due to the faster degradation nature of the autologous bone graft. The newly formed bone surrounding the graft material was also observed in the DBB and BioCaP group (Figures 5C1,D1). However, the area covered by newly formed bone in DBB or BioCaP group was less compared to BMP2.BioCaP or autologous group. This indicates the lack of osteoinductive properties in DBB or BioCaP grafts. A robust amount of new bone was formed surrounding the graft materials and in spaces between the graft materials in BMP2-sur.BioCaP and BMP2-int.BioCaP groups (Figures 5E1,F1). The results of histology images are per the results of micro-CT images (Figures 3–5).

### Active Bone Regeneration and Bone Remodeling in BMP2.BioCaP Graft Groups

**Figure 6:**
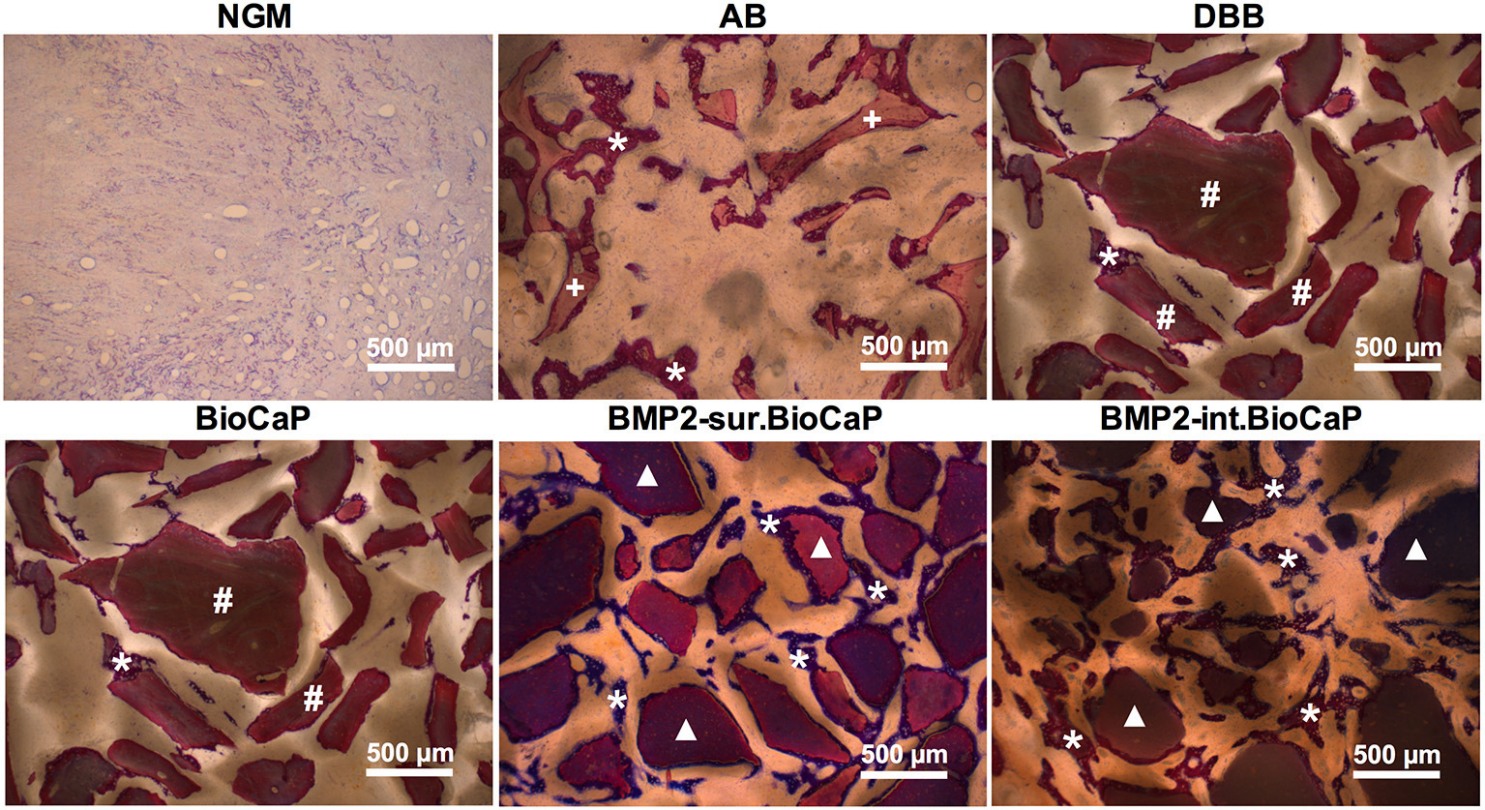
Histological images in higher magnification showing newly formed bone and grafted biomaterials in the defect site after 4 weeks of grafting. White stars: remaining graft materials; +: remaining autologous bone; #: remaining DBB.

**Figure 7:**
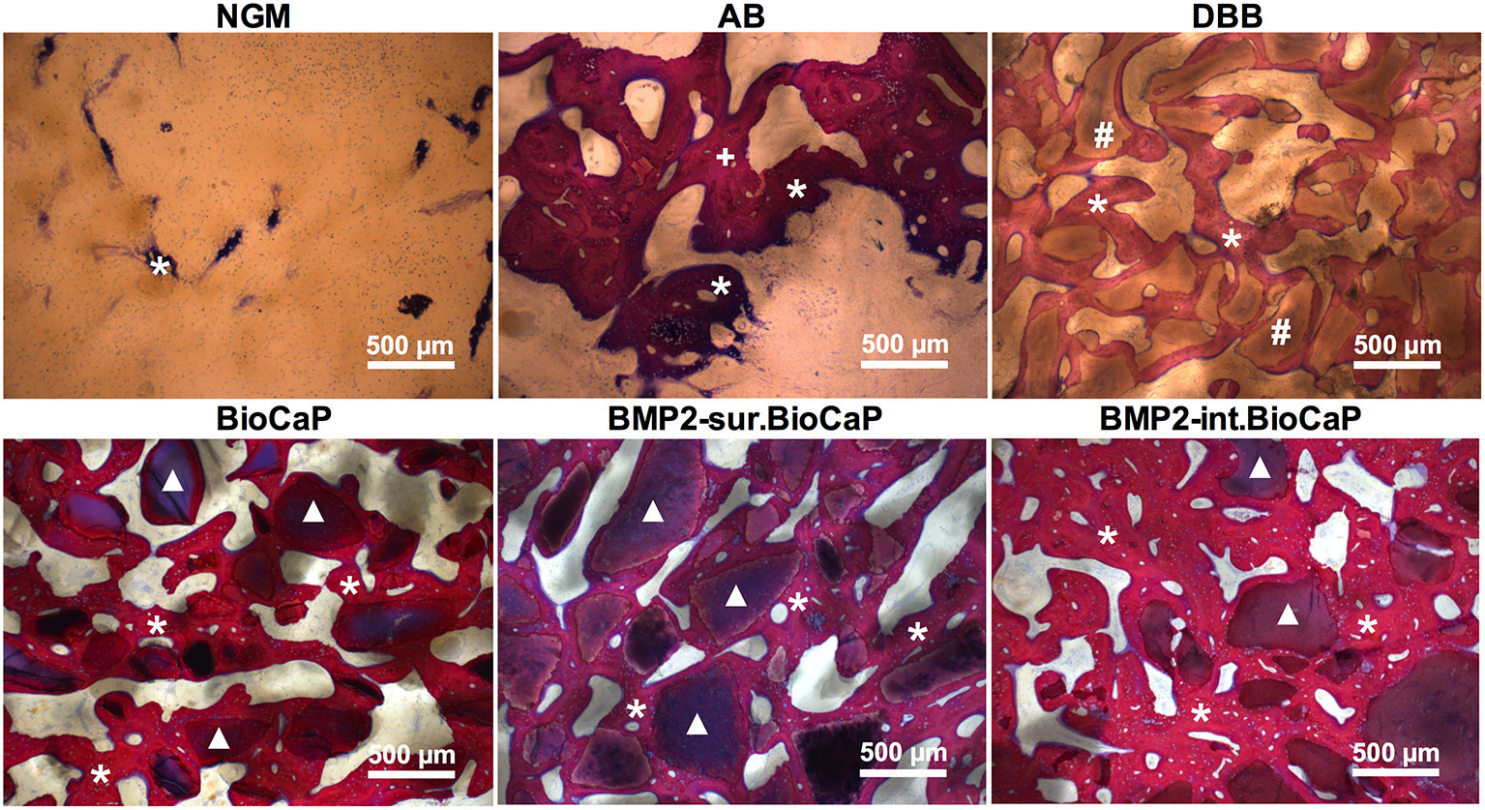
Histological images in higher magnification showing newly formed bone and grafted biomaterials in the defect site after 8 weeks of grafting. White stars: newly formed bone; +: remaining autologous bone; #: remaining DBB.

Figure 6 represents the higher magnification histological images of bone defects at week 4. No graft group was mainly covered with fibrous tissues at week 4. Robust osteogenesis around the graft and in space between the grafts was observed in the autologous bone graft group. DBB and BioCaP group showed sporadic thin layers of newly formed bone around the graft materials and in space between the grafts. Both the BMP2.BioCaP graft groups showed robust bone regeneration throughout the defect compared to the DBB or BioCaP group. Figure 7 represents the higher magnification histological images of bone defects at week 8. Newly formed bone was hardly observed in the no graft group. The autologous graft group showed big areas of newly formed bone but no graft material was left. BMP2-sur.BioCaP and BMP2-int.BioCaP groups showed phenomenal bone regeneration compared to DBB or BioCaP group. All the groups with higher bone regeneration showed the woven bone. BMP2.BioCaP group showed the higher area with trabecular bone appearance, indicating active bone remodeling of woven bone in these groups. In both time points (week 4 and 8), it was difficult to recognize coating around the BMP2.BioCaP, indicating complete degradation of coating possibly by the effect of biological fluid or/and multinucleated giant cells.

Further high-resolution magnification of histological images indicated active bone regeneration and bone remolding activities at week 4 and 8. Representative histological images of BMP2.BioCaP grafts showed the presence of abundant cells, mainly mononuclear immune cells in the space between the graft materials (Supplementary Figure 1). At week 8, the number of mononuclear immune cells was reduced drastically. Chondrocytes and hypertrophic chondrocytes-like cells were observed in the newly formed bone area indicating the active endochondral ossification. Lining osteoblasts were observed on the surface of the newly formed bone. Moreover, osteocytes in lacunae and osteoids were observed inside the newly formed bone at week 8. Similarly, active multinucleated cells on the surface of newly formed bone indicate the active bone remodeling process during bone defect healing (Supplementary Figure 2). Bone marrow-like structures and blood vessel-like structures were observed in the space between the graft materials. Osteoclast-resorbed and bio-actively-degraded irregular edges were observed in the DBB, BioCaP, or BMP2.BioCaP grafts.

### Quantitative Micro-CT and Histomorphometric Analysis

**Figure 8:**
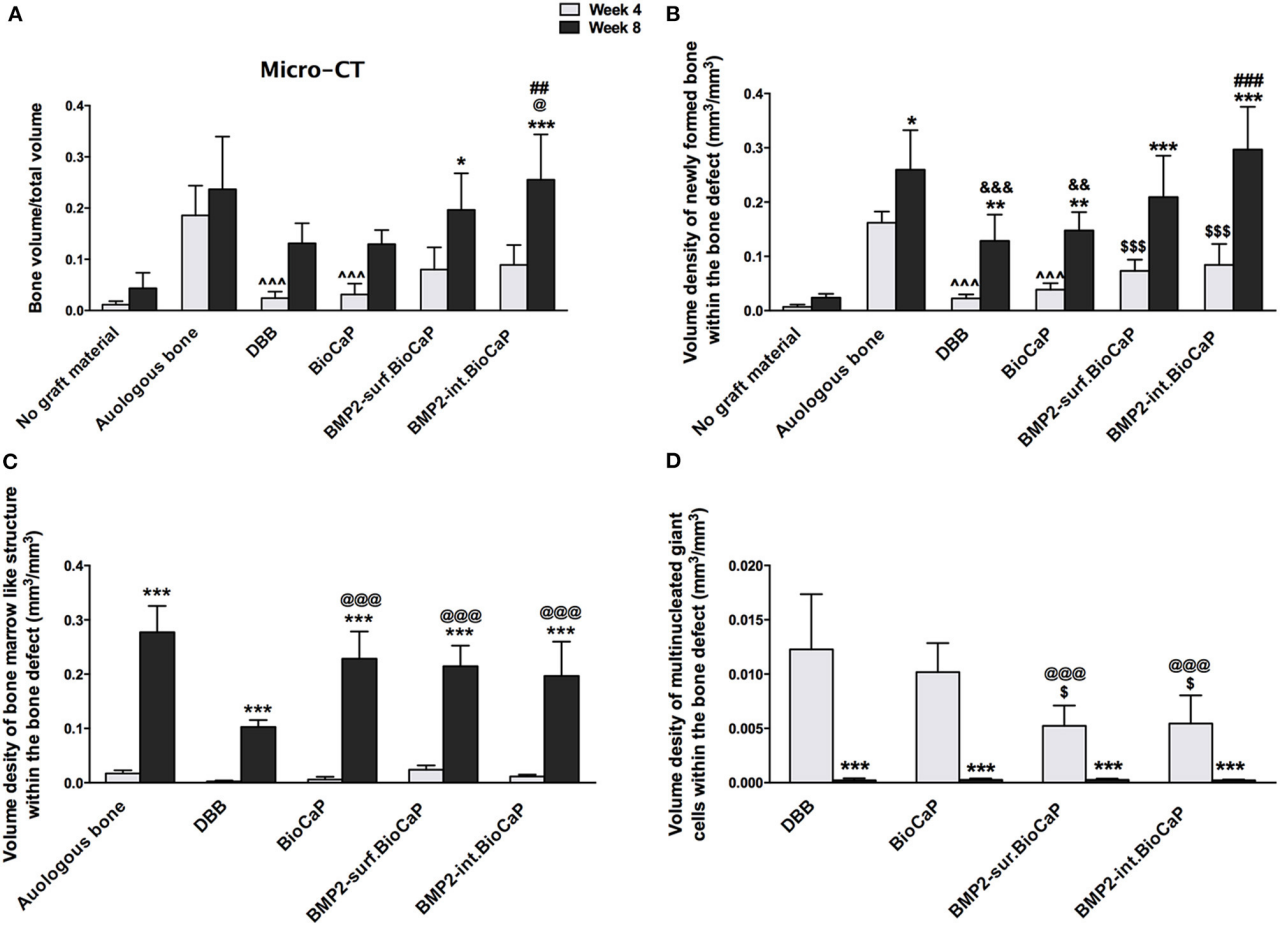
**(A)** Bone volume/Total volume of newly formed bone analyzed from micro-CT data. Volume density of: **(B)** newly formed bone, **(C)** bone marrow-like structures, and **(D)** multinucleated giant cells analyzed form histological images. Data are presented as mean ± SD (*n* = 6). Significant difference compared to respective week 4-group, *p < 0.05, **p < 0.01, and ***p < 0.001; compared to BioCaP week 4-group, ^*$*^*p* < 0.05 and ^*$$$*^*p* < 0.001; compared to DBB week 8-group or week 4-group, ^@^*p* < 0.05, ^@*@@*^*p* < 0.001; compared to BioCaP week 8-group, ^*##*^*p* < 0.01, ^*###*^*p* < 0.001; compared to autologous bone week 4-group, ^∧∧∧^*p* < 0.001; compared to autologous bone week 8-group, ^&&^*p* < 0.01, ^&*&&*^*p* < 0.001.

Quantitative micro-CT analysis was performed to analyze bone volume/total volume in the site of the defects at week 4 and 8 (Figure 8A). BMP2-int.BioCaP group at week 8 showed higher bone volume/total volume compared to DBB and BioCaP grafts. Bone volume/total volume in BMP2-sur.BioCaP and BMP2-int.BioCaP groups was to a similar extent to that of autologous bone graft group. Histomorphometric analysis was performed to quantify the volume density of newly formed bone, bone marrow-like structure, blood vessel-like structure, multinucleated giant cells, and graft material degradation % in the different materials grafted bone defects. Except in the no graft group, all other groups showed a higher volume density of newly formed bone at week 8 compared to the respective group at week 4 (Figure 8B). This result revealed that bone formation significantly increased with the implantation duration. The autologous bone group showed a significantly higher volume density of newly formed bone compared to DBB or BioCaP group at week 4 and 8. BMP2-sur.BioCaP and BMP2-int.BioCaP group showed 3.3-, and 3.7-fold higher volume density of newly formed bone respectively at week 4 compared to the DBB group. BMP2-sur.BioCaP and BMP2-int.BioCaP group had a similar degree volume density of newly formed bone with the autologous bone graft group at week 4 and 8. BMP2-int.BioCaP but not BMP2-sur.BioCaP enhanced bone regeneration compared to the BioCaP group at week 8, indicating the better osteoinductive properties of BMP2-int.BioCaP grafts. The results of quantitative micro-CT analysis on newly formed bone were in accordance with the results from the histomorphometric analysis (Figures 8A,B).

There was no bone marrow-like structure in the no graft group at week 4 and 8. In all other groups, the quantitative evaluation revealed a robust increase in the volume density of bone marrow-like structures at week 8 compared to the respective group at week 4. There was no difference in the volume density of bone marrow-like structures among the groups at week 4 (Figure 8C). The autologous graft group showed the highest volume density of bone marrow-like structures at week 8. BioCaP, BMP2-sur.BioCaP, or BMP2-int.BioCaP group showed a higher volume density of bone marrow-like structures compared to the DBB group at week 8. BioCaP and BMP2.BioCaP groups showed an almost similar degree of the volume density of bone marrow-like structures to the autologous group at week 8 (Figure 8C).

There was no significant difference in the volume density of newly formed blood vessel-like structures among the groups at week 4 or 8 (Supplementary Figure 3A). Multinucleated giant cells were hardly visualized in the no graft or autologous group at week 4 and 8. In the remaining four groups, the volume density of multinucleated giant cells significantly was reduced at week 8 compared to the respective group at week 4 (Figure 8D). BMP2-sur.BioCaP or BMP2-int.BioCaP group showed a less volume density of multinucleated giant cells compared to DBB or BioCaP group at week 4, indicating less immunogenicity of BMP2.BioCaP grafts. In the autologous group, there was excessive degradation of graft materials at week 4 and almost no graft material was left at week 8. Among the other groups, there was no difference in the graft materials degradation % at week 4 and week 8 (Supplementary Figure 3B). The results of this study indicate the osteoconductive, robust osteoinductive, less immunogenic, and phenomenal bone regenerative potential of BMP2-sur.BioCaP and BMP2-int.BioCaP grafts. BMP2-int.BioCaP graft showed better bone defect healing potential than BMP2-sur.BioCaP.

## Discussion

Bone grafts are the second most implanted grafts due to the increase in the cases of bone defects mainly caused by trauma, old-age, and disease-related bone damages. Due to limited sources and other shortcomings of the autografts, allografts, or xenografts, the tissue-engineered biomaterials are being developed as an optional source of the bone grafts for clinical use. Advances in biomaterials had led to the development of osteogenic/angiogenic growth factors loaded tissue-engineered bone grafts. However, osteogenic bioactivity, biosafety, and local/systemic adverse effects are still key issues of synthetic bone grafts that need to be addressed. In this study, we tested the bone defect healing potential of biomimetically fabricated BMP2.BioCaP grafts (BMP2-sur.BioCaP and BMP2-int.BioCaP) in sheep humerus and femoral bone defects.

BMP2-sur.BioCaP and BMP2-int.BioCaP grafts doped ~1000 and 500-fold less BMP2, respectively than used in clinics for bone regeneration (Block and Achong, 2006; Liu et al., 2017). Both Bone grafts showed robustly higher bone defect-healing potential compared to clinically used DBB grafts. The bone defect healing potential of BMP2.BioCaP grafts was as effective as that of the autologous bone graft. Although, BMP2-int.BioCaP graft doped half the amount of BMP2 compared to BMP2-sur.BioCaP graft, the bone defect healing potential of BMP2-int.BioCaP was even robust. Different ways of BMP2 doping did not affect the surface morphology and degradation properties of the graft materials. The BMP2.BioCaP grafts showed less immunogenicity compared to BioCaP or DBB bone grafts. Bone defect sites were healed well without any significant wound complication, signs of inflammation, or adverse host tissue reactions. Our findings indicate the possibility of clinical application of biomimetically fabricated BMP2.BioCaP grafts.

Bone graft biomaterials directly interact with the precursor cells and affect cellular activity and differentiation (Gao et al., 2017). Similarly, the graft material-mediated immunogenicity modulates immune cells' function regulating inflammation in bone defect milieu (Chen Z. T. et al., 2016). Chemical remnants in grafts used for material synthesis can induce graft-related immunogenicity. Biomimetically synthesized biomaterials minimize the immunogenicity and graft material-related local/systemic adverse effects (Greenwald et al., 2001; Fernandez-Yague et al., 2015; Sun et al., 2019; Green et al., 2020). In this study, BMP2.BioCaP grafts were synthesized biomimetically as we reported previously (Liu et al., 2014, 2017; Wang et al., 2017, 2019). BMP2.BioCaP grafts showed less immunogenicity compared to DBB or BioCaP grafts as indicated by the fewer numbers of multinucleated giant immune cells around in BMP2.BioCaP grafts. Foreign body multinucleated giant cells resorb the graft materials and also remodel newly formed bone (Milde et al., 2015; Barbeck et al., 2017). Higher immunogenicity of graft materials triggered the formation of multinucleated cells and inflammatory cascade (Barbeck et al., 2017). In this study, the volume density of multinucleated giant cells was significantly higher in the DBB or BioCaP group compared to BMP2.BioCaP groups at week 4. This result is in accordance with the result from the previous study of ectopic and *in-situ* implantation of BMP2.BioCaP grafts (Liu et al., 2014, 2017). Since, there was no significant difference in the graft materials degradation among the DBB, BioCaP, or BMP2.BioCaP groups, the higher volume density of multinucleated giant cells in DBB or BioCaP might be associated with a higher degree of inflammation.

BMP2.BioCaP grafts give slow and sustained release of low dose BMP2 in the defect area and this release is mainly cellular activity-dependent (Liu et al., 2017). The robust bone regenerative and less immunogenic properties of BMP2.BioCaP grafts might be the effect of this BMP2 release phenomenon. Moreover, slow and sustained release of low dose BMP2 in the defect area might be responsible for the well-healed bone defects without any significant wound complication, signs of inflammation, or adverse host tissue reactions.

Active bone regeneration and bone remodeling are essential for the graft-mediated effective bone defect healing. Recruitment of osteogenic/endothelial precursor cells and immune cells to the graft material is inevitable for bone regeneration and remodeling process (Shi et al., 2016). BMP2.BioCaP graft group showed higher numbers of progenitor cells and immune cells at week 4. The density of immune cells and progenitor cells around the graft materials and newly formed bone was drastically reduced during week 8 of bone defect healing compared to week 4. This indicates the importance of early recruited progenitor cells and immune cells on bone defect healing. During bone defect healing, precursor cells undergo chondrogenic differentiation forming the cartilaginous soft callus that subsequently transfers to the mineralized-bone matrix via endochondral ossification (Nilsson Hall et al., 2020). In this study, we observed chondrocytes and hypertrophic chondrocyte-like structures in or nearby the newly formed bone. This result indicates the endogenous bone regenerative potential of BMP2.BioCaP grafts via endochondral ossification. A prominent number of osteoblast-like lining cells and multinucleated osteoclast-like cells on the surface of the newly formed bone in the BMP2.BioCaP grafts group indicate active bone remodeling. Moreover, A similar extent of the volume density of newly formed bone, bone marrow-like structure, and blood vessel-like structures in BMP2.BioCaP graft groups compared to that in autologous graft group suggests a highly active bone regenerative and bone remodeling potential of BMP2.BioCaP grafts during bone defect healing.

In this study, we analyzed the bone defect healing potential of BMP2.BioCaP grafts in a large animal model. Furthermore, we compared the bone defect healing potential of BMP2.BioCaP graft with the gold standard autologous bone graft and clinically used DBB bone graft. The bone defect healing potential of BMP2.BioCaP grafts was robustly superior compared to BioCaP or DBB graft, and as effective as that of autologous bone graft. Week 4 is the initial stage of new bone formation in the defects of the large animal model as shown by presence of precursor cells and a small amount of newly formed bone in BMP2.BioCaP and autologous bone graft groups. At week 8, the newly formed in mainly woven bone, which requires to undergo the major remodeling for 6–12 months to convert into real load-bearing bone (Hettwer et al., 2019). The use of femoral bone defect in 4 years old adult sheep model allowed us to investigate the biocompatibility, degradation, and bone regenerative potential of the BMP2.BioCaP grafts (Nuss et al., 2006). However, the bone volume, osteoid volume, and mineral apposition rate of 9–10 years old sheep resembles those of 60–70 years humans (Turner, 2001). Therefore, the preclinical study, using segmental bone defect in old age sheep analyzing the major bone remodeling for 6–12 months (Reichert et al., 2009; Hettwer et al., 2019), is further required to understand the bone defect healing potential of BMP2.BioCaP grafts. Early mechanical stimulation is necessary for timely bone defect healing (Tufekci et al., 2018). The current study lacks the role of mechanical stimulation in BMP2.BioCaP-mediated bone defect healing in sheep. The limitation of BMP2.BioCaP graft is the small size of each graft granule (0.25–1.0 mm) that is not suitable to graft in extra-large bone defect and load-bearing site such as segmental bone defects. It is difficult to custom design BMP2.BioCaP granules-based bone graft with a specific shape and certain mechanical strength. However, the biomimetic coating of BMP2.BioCaP on the surface of CaP-based 3D-printed scaffolds could osteogenically functionalize the large size bone grafts designed for critical size bone defects (Lin et al., 2020).

## Conclusions

Biomimetically fabricated BMP2.BioCaP grafts reduce the clinical dose BMP2. BMP2.BioCaP grafts showed biocompatibility and robust bone regenerative potential in the ectopic model and in *in-situ* bone defects of small and large animals. Our results warrant the pre-clinical studies in critical size segmental bone defect of large animals to access the bone defect healing potential of BMP2.BioCaP grafts.

## Data Availability Statement

The original contributions presented in the study are included in the article/Supplementary Materials, further inquiries can be directed to the corresponding authors.

## Ethics Statement

The animal study was reviewed and approved by the Ethical Committee of School of Stomatology, Zhejiang University.

## Author Contributions

TL, WF, JP, and YL: study concept, design, and data interpretation. YL and TL: data acquisition and analysis. TL and WF: experiment and manuscript writing. GW: BMP2.BioCaP preparation and manuscript editing. All authors read and approved the submitted version.

## Conflict of Interest

The authors declare that the research was conducted in the absence of any commercial or financial relationships that could be construed as a potential conflict of interest.

## Supplementary Material

Supplementary Figure 1 Histological images showing active bone regeneration process during 4 and 8 weeks of grafting in the BMP2-sur.BioCaP group. White star: newly formed bone; white triangle: remaining graft; black arrow: mononuclear immune cells; yellow arrow: chondrocytes and hypertrophic chondrocytes-like cells; green arrow: osteoblast lining cells.

Supplementary Figure 2 Histological images showing active multinucleated giant cells, bone marrow-like, and blood vessel-like structures. White star: newly formed bone; white triangle: remaining graft; black arrow: active osteoclasts; yellow arrow: chondrocytes and hypertrophic chondrocytes-like cells; green arrow: osteoblast lining cells; pink arrow: resorbed graft material by active osteoclasts; M: bone marrow-like structure; V: blood vessel-like structure.

Supplementary Figure 3 Quantitative analysis of volume density of blood vessels like structure **(A)**, and graft material degradation (%) **(B)**. Data are presented as mean ± SD (*n* = 6).
