# Supplementary figures and images for: Low Dose BMP2-Doped Calcium Phosphate Graft Promotes Bone Defect Healing in a Large Animal Model
Source: Front Cell Dev Biol. 2021 Jan 21;8:613891. doi: 10.3389/fcell.2020.613891 (PMC7858265; doi:10.3389/fcell.2020.613891)

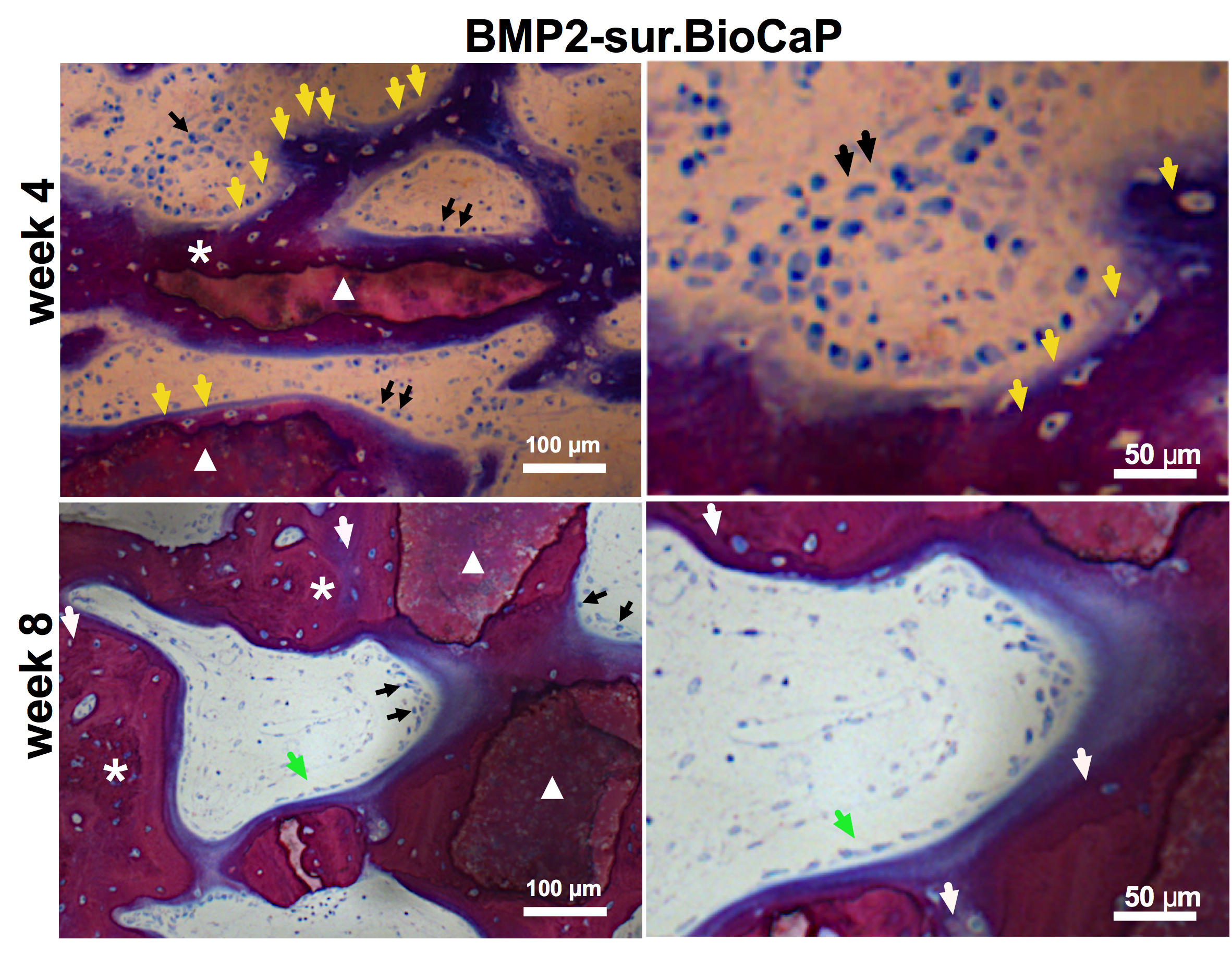

Supplement: Supplementary Figure 1 — Histological images showing active bone regeneration process during 4 and 8 weeks of grafting in the BMP2-sur.BioCaP group. White star: newly formed bone; white triangle: remaining graft; black arrow: mononuclear immune cells; yellow arrow: chondrocytes and hypertrophic chondrocytes-like cells; green arrow: osteoblast lining cells. [file Image_1.TIFF]

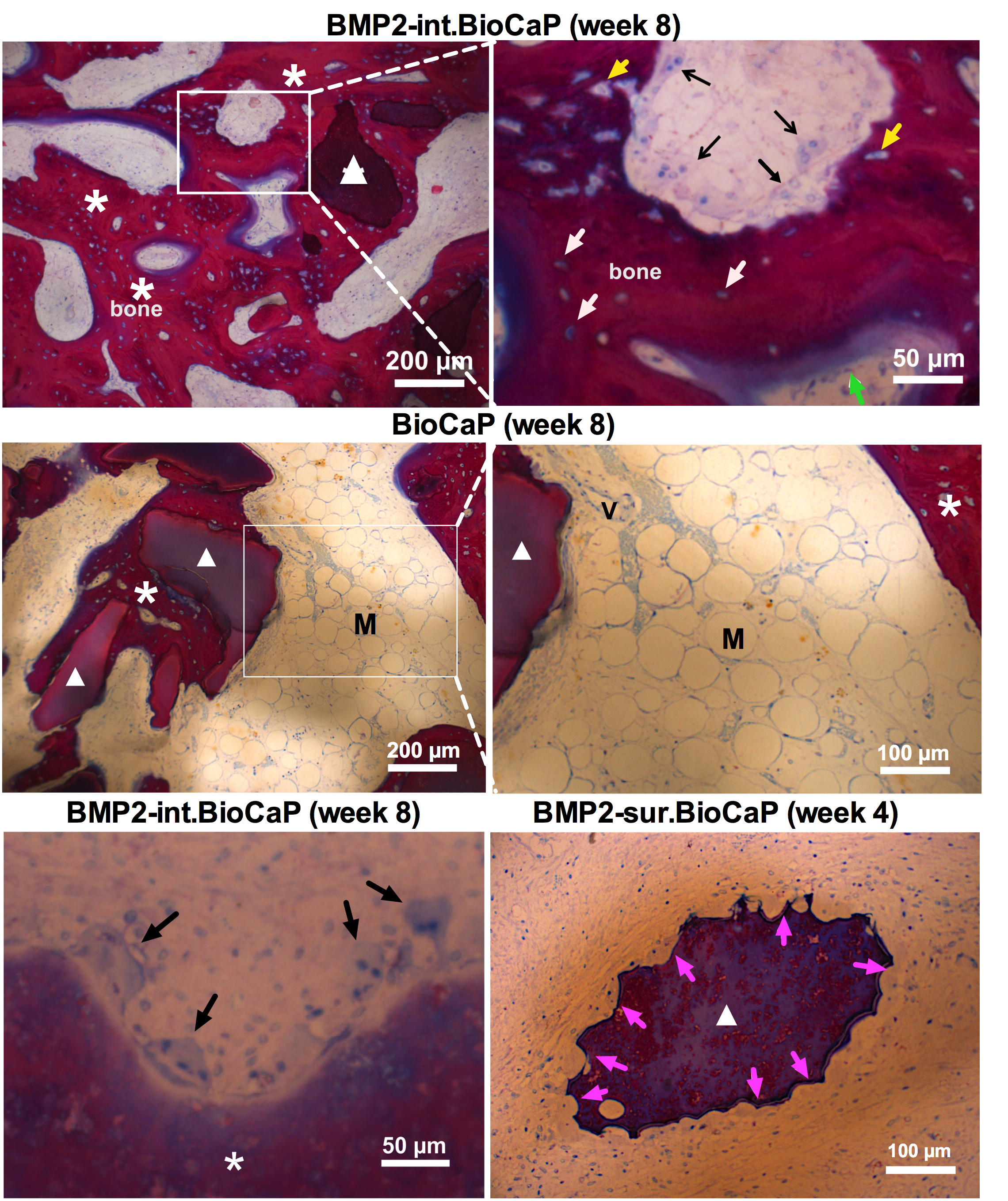

Supplement: Supplementary Figure 2 — Histological images showing active multinucleated giant cells, bone marrow-like, and blood vessel-like structures. White star: newly formed bone; white triangle: remaining graft; black arrow: active osteoclasts; yellow arrow: chondrocytes and hypertrophic chondrocytes-like cells; green arrow: osteoblast lining cells; pink arrow: resorbed graft material by active osteoclasts; M: bone marrow-like structure; V: blood vessel-like structure. [file Image_2.TIFF]

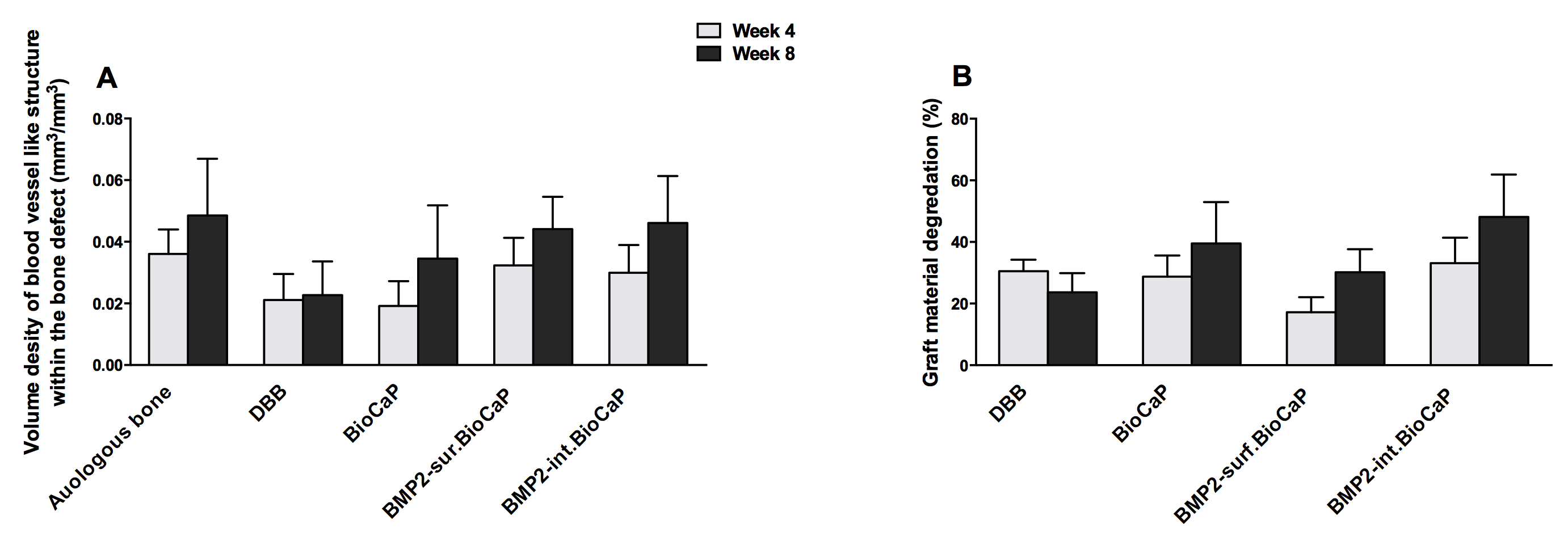

Supplement: Supplementary Figure 3 — Quantitative analysis of volume density of blood vessels like structure (A), and graft material degradation (%) (B). Data are presented as mean ± SD (n = 6). [file Image_3.TIFF]
